# One year of COVID-19 pandemic: a cross sectional study on teaching oral and maxillofacial surgery

**DOI:** 10.1186/s13005-021-00304-z

**Published:** 2021-12-18

**Authors:** Anna Bock, Florian Peters, Philipp Winnand, Kristian Kniha, Marius Heitzer, Martin Lemos, Frank Hölzle, Ali Modabber

**Affiliations:** 1 Department of Oral and Maxillofacial Surgery, University Hospital RWTH Aachen, Pauwelsstrasse 30, D-52074 Aachen, Germany; 2 Audiovisual Media Center, Medical Faculty, RWTH Aachen University, Pauwelsstraße 30, D-52074 Aachen, Germany

**Keywords:** COVID-19, Dental education, Digitalization, Pandemic, Survey

## Abstract

**Background:**

The pandemic has challenged educational institutions to catalyze digitalization and rapidly develop online teaching formats. The aim of the study was to evaluate the teaching offered for oral and maxillofacial surgery at our university during the pandemic and to investigate the students’ perceptions of the current situation.

**Methods:**

A 38-item questionnaire with five sections (demographic information, lectures, internships, e-learning, and pandemic-related solutions/effects) was created online. Most questions were answered on a 10-point Likert scale, with 1 indicating “fully agree/positive” and 10 indicating “totally disagree/negative.” The remaining questions were either answered with yes/no, percent value, or open-ended text responses. All 3rd-5th year dental students were invited to voluntarily participate and were sent a link by email in a general mail shot.

**Results:**

A total of 63.7% of the participants had no prior experience with online courses before the pandemic. The students stated that the change from face-to-face to online teaching worked very well in the last two semesters (mean = 2.73, standard deviation = 2.05). Overall, the pandemic had a rather positive influence on the acquisition of theoretical skills and a negative influence on the acquisition of practical skills (*p* < 0.0001). The evaluation showed that, compared to other dental clinics at our university, the department for oral and maxillofacial surgery was well prepared for the pandemic.

**Conclusion:**

Digitalization of oral and maxillofacial surgery teaching in dental education is possible but depends on the institution’s preparatory work and technological possibilities. The students declared a high acceptance of digital learning formats and indicated an increased motivation to learn due to e-learning. The pandemic’s influence on the students’ education was rated ambivalent.

**Supplementary Information:**

The online version contains supplementary material available at 10.1186/s13005-021-00304-z.

## Background

One year after the start of the coronavirus disease 2019 (COVID-19) pandemic in Europe, the disease has revealed a massive impact on all aspects of life [1–3]. Restrictions like curfews, quarantine or closures of public places and educational institutions to control the rate of infections have been imposed. These restrictions have also a significant effect on academic teaching as it has been established in recent years. When face-to-face classes were temporarily impossible, teaching methods suddenly shifted toward online. Lectures and seminars were replaced by videos and Zoom lectures/seminars, and internships were canceled or replaced by other e-learning formats. Online teaching had to develop rapidly, and therefore the pandemic worked as a catalyzer for digitalization at universities [4, 5]. Teachers were often faced with major challenges, as faculties sometimes required digital conversion of teaching within a few days or weeks [6]. Some academic institutions were completely unprepared, while others had already done good preparatory work for online teaching.

At our university, the teaching of oral and maxillofacial surgery is spread over 2.5 years (3rd-5th year) and is divided into 4 series of lectures and 2 internships. To improve the quality of the teaching offer, new teaching methods and e-learning formats have been implemented for several years. For example, a blended learning concept has been applied to the main oral and maxillofacial surgery lecture. Students can prepare for and follow up on the lecture with the help of an e-learning program. Besides text elements, the e-learning program contains several surgical videos, photos, and a quiz for self-assessment [7]. Further, an e-learning format following the flipped classroom principle for the oral surgery internship has been introduced. An e-learning program containing the educational content with additional didactically prepared oral surgery videos has been developed. Here, students are called upon to prepare the learning content at home in order to apply the newly learned knowledge directly in the internship. This method has been confirmed to be effective in improving knowledge and competence among dental students [8]. In addition, students have confirmed their high satisfaction with the blended learning approaches [7, 8]. The implementation of the valuable digital teaching infrastructure took almost 5 years and was supported by various funds.

Since all face-to-face classes were cancelled at the beginning of the pandemic, all teaching had to be switched from face-to-face/hybrid to online courses. With the introduction of hygiene concepts and vaccinations, the internships could take place again. The lectures remained online events.

The aim of the study was to evaluate the teaching for oral and maxillofacial surgery at our university during the pandemic and to investigate the students’ perceptions of the current situation.

## Methods

### Questionnaire design and distribution

A 38-item questionnaire was developed following a literature search on the current effects of COVID-19 on dental education. The survey consisted of five sections: demographic information, lectures, internships, e-learning, and the pandemic-related solutions/effects. Most questions were answered on a 10-point Likert-scale, with 1 indicating “fully agree/positive” and 10 indicating “totally disagree/negative.” The remaining questions were either answered with yes/no, percent value, or open-ended text responses (see supplementary material). Three professionals validated and approved the final questionnaire. The survey was created using Lime Survey, an online survey software (LimeSurvey GmbH, Hamburg, Germany), and distributed with an anonymous link by email. The survey was accessible online for three weeks (April 15 to May 7, 2021).

### Participants, consent, and ethical considerations

All 3rd-5th year dental students were invited via email by the faculty’s secretary to voluntarily participate in this cross-sectional study. The participants were informed that the collected data were to be used for the research and would be completely anonymous. Informed consent was obtained prior to starting the survey. All methods were carried out in accordance with the relevant guidelines and regulations. The local Ethics Commission approved the study (EK 151/21).

### Statistics

The obtained data were arranged using MS Office Excel 2019® (Microsoft Corporation, Redmond, Washington, USA). Statistical analyses were performed using GraphPad Prism 6 software (GraphPad Software, San Diego, California, USA). The continuous variables were reported as mean and standard deviation (SD). Normal distribution was checked through a D’Agostino-Pearson normality test in the omnibus K2 variant. The unpaired t-test was used to compare the evaluated aspects. *P* < 0.05 was considered statistically significant. One-way ANOVA was used to analyse the influence of demographic aspects on the adaption to the pandemic.

## Results

### Demographics

A total of 117 out of 149 students (78.5%) participated in this study. There were 15 dropouts due to incomplete questionnaire answers. Out of the 102 participants, 70 were female, 30 male and 2 were other. In terms of age, 6% were ≤ 21 years old, 66.7% were 22-25 years old and 27.3% were ≥ 26 years old. Out of the respondents, 17.5% were in their 3rd year, 37.1% were in their 4th year, and 45.4% were in their 5th year. The demographics (gender, age) did not have a significant influence on the students’ education overall during the pandemic (gender: *p* = 0.597, age: *p* = 0.321).

### Lectures and internships

The students were asked about their participation in lectures and internships: 81.8% participated ≥81, 15.2% participated 61-80 and 3% participated only 41-60% in the online courses during the past year. The participation in the online courses was voluntary.

**Fig. 1:**
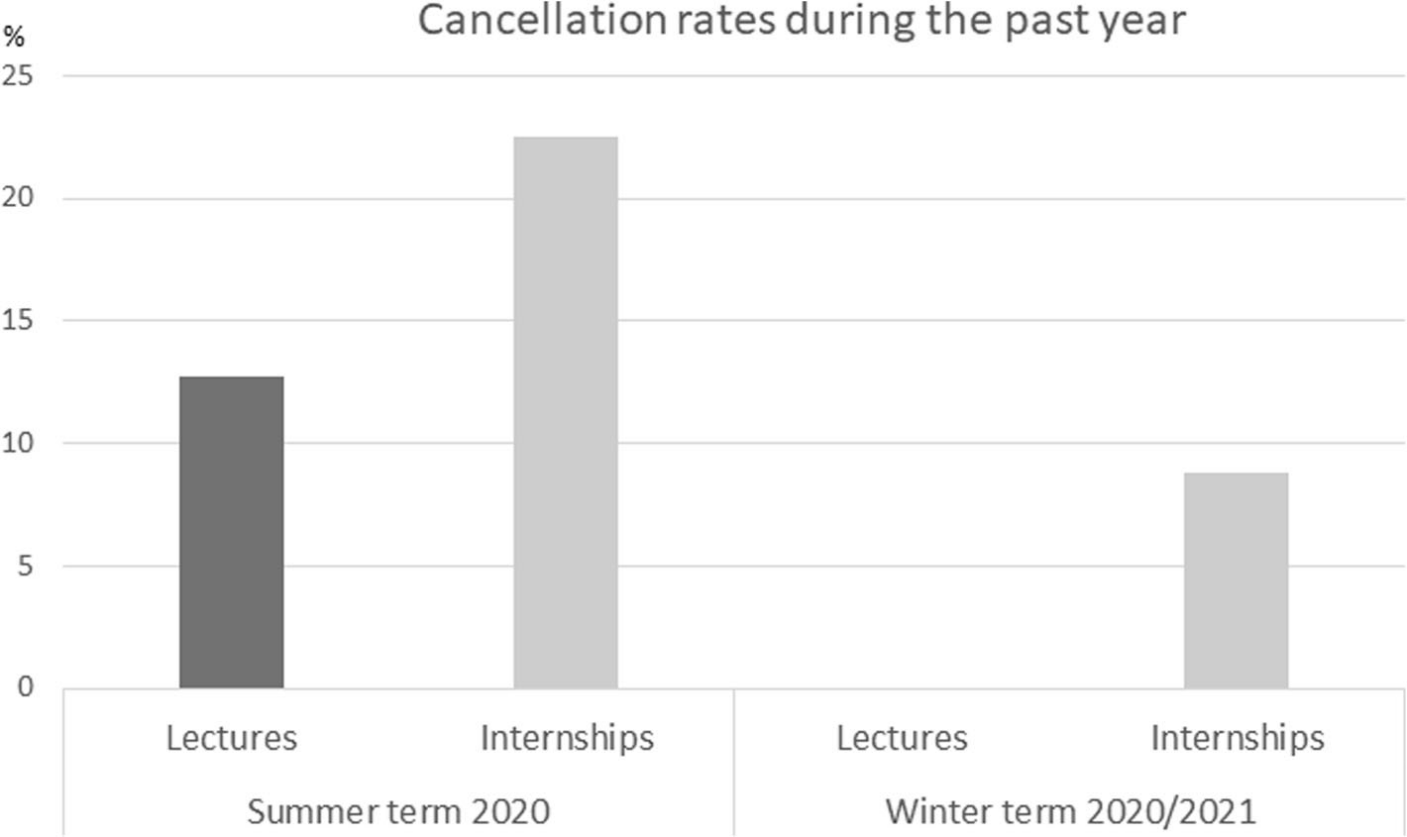
Cancelation rates during the last two semesters. In the second semester during the pandemic, the cancelation rates of the internships were reduced, and no lectures were cancelled

**Fig. 2:**
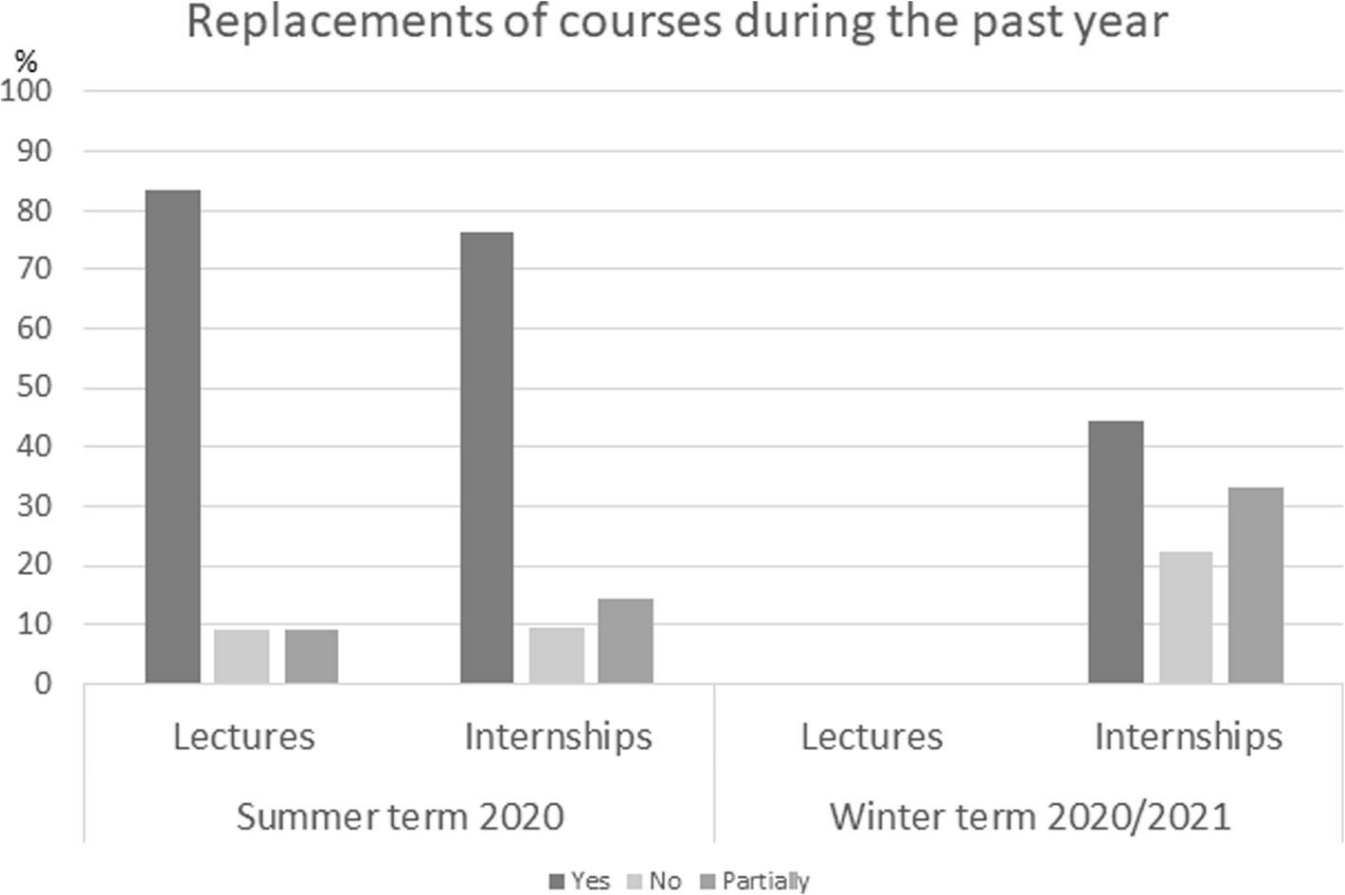
In the case of cancelation, most courses could be replaced or caught up with

The cancelation rates are shown in Fig. 1. Regarding cancelation, most students stated that ≤25% of the courses were canceled. In the event of cancellation, the courses with the same educational content were postponed to another day and time. The course replacements are shown in Fig. 2.

**Fig. 3:**
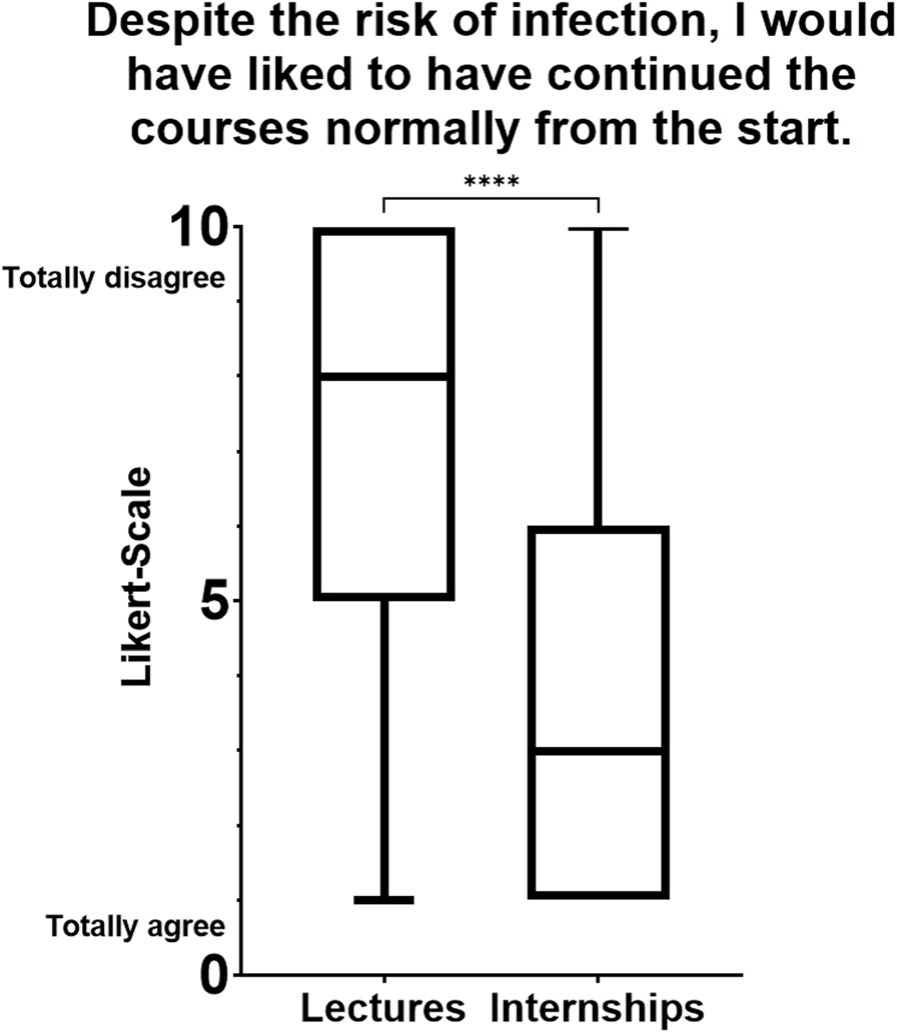
Most students stated that they would have liked to have continued their internships normally from the start, despite the risk of infection. Compared to the answers about continuing the lectures normally from the start, the difference was significant (*p* < 0.0001)

Regarding the students’ opinions on the continuation of courses from the start of the pandemic, most students would have liked to continue the internships normally but not the face-to-face lectures, as shown in Fig. 3.

### E-learning

Of the participants, 63.7% had no prior experience with online courses before the pandemic, 17.6% stated that they had prior experience, and 18.6% did not answer the question. The other results of the e-learning evaluation are shown in Table 1.

**Table 1:** Evaluation of e-learning. All aspects were rated on a 10-point Likert scale, with 1 indicating “totally agree/positive” and 10 “totally disagree/negative”

	Mean Score	Standard deviation
Thanks to the extensive digital range of oral and maxillofacial surgery online (Emedia Skills Lab) developed in recent years, the MKG was well prepared for the pandemic.	2.39	2.27
In my opinion, the effectiveness of knowledge transfer through online events has improved significantly compared to face-to-face events.	3.88	2.61
In the last two semesters there were often technical problems with online teaching.	6.47	3.02
The change from face-to-face to online teaching worked very well in the last two semesters.	2.73	2.05

### Pandemic-related solutions/effects

The results of the evaluation of the pandemic- related solutions and effects are shown in Table 2.

**Table 2:** Evaluation of pandemic-related solutions and effects. All aspects were rated on a 10-point Likert scale, with 1 indicating “totally agree/positive” and 10 indicating “totally disagree/negative”

	Mean Score	Standard deviation
The organization of the switch from face-to-face to online teaching was very good.	2.71	2.09
How has the pandemic affected your student education overall?	5.94	2.33
The new digital learning conditions are very stressful for me.	7.57	2.53
I am concerned that I will not pass some courses because of the pandemic conditions.	5	3.34
I am concerned that my exam grades will change due to the pandemic conditions.	6.86	2.24
I would like to keep the digital courses (e-learning programs and digital lectures) in the future.	2.67	2.47
In the future, I prefer to further reduce face-to-face events and replace them with online courses.	4.48	2.96
The online courses motivated me to learn more.	3.67	2.64
The pandemic made it easier for me to focus on my studies because there was less distraction from leisure activities.	6.35	3.18

Most students stated that the new digital teaching formats were not stressful for them (mean = 7.57, SD = 2.53). Students declaring the opposite stated in the open-ended question that they were stressed because they had difficulties concentrating at home and that there was a lack of time in hybrid courses when switching from online to face-to-face classes.

**Fig. 4:**
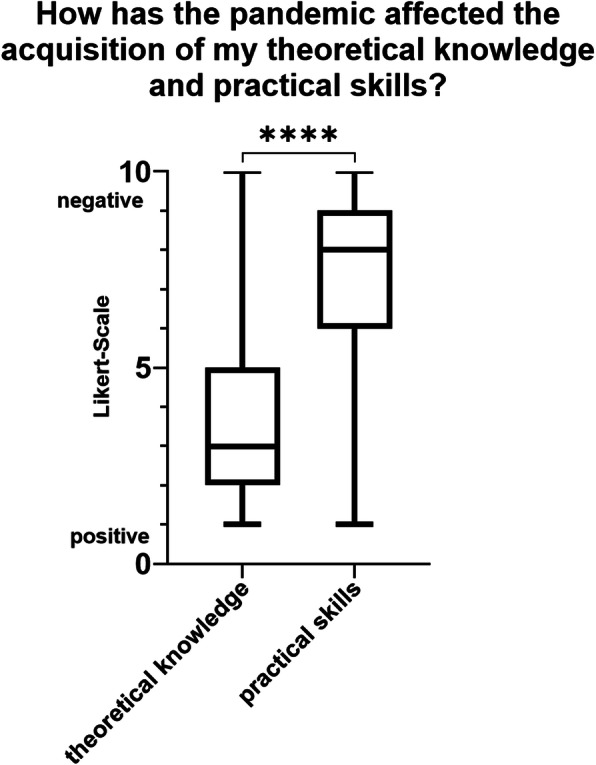
Students evaluated that the pandemic had a somewhat positive influence on the acquisition of theoretical skills and a rather negative influence on the acquisition of practical skills. The difference was significant (*p* < 0.0001)

The assessment of the students with regard to the acquisition of theoretical knowledge and practical skills showed that the effect on theoretical knowledge gain tended to be a positive while the effect on practical skills was negative (see Fig. 4). The difference was significant (*p* < 0.0001).

In terms of which digital teaching format the participants preferred, multiple answers were possible. 43.16% of the students stated that they preferred live lectures, 70.53% preferred recorded lectures and 48.42% preferred e-learning programs.

Further, the students highlighted in the open-ended responses that, compared to other dental clinics at our university, the department for oral- and maxillofacial surgery was well prepared for the pandemic. The organization of the different courses was very well structured, and the switch from face-to-face to online teaching was successful.

## Discussion

The pandemic has forced universities to completely digitalize existing teaching formats. Some academic institutions were completely unprepared, while others had already prepared well for online teaching. This cross-sectional study aimed to evaluate the teaching offered for oral and maxillofacial surgery at our university during the pandemic and to investigate the students’ perceptions of the current situation.

Overall, the participants evaluated the teaching offer for oral and maxillofacial surgery to be fairly good. The students highlighted that, compared to other dental clinics at our university, the department for oral- and maxillofacial surgery was well prepared for the pandemic. The participants confirmed that the previously developed blended learning concepts contributed to the good preparation, and the change from face-to-face to online teaching worked very well. During the first pandemic year, only minor parts of the lectures and the internships were canceled, and, in the case of cancelation, it was possible to replace the courses.

Most students stated that they would have liked to have continued their internships normally from the start, despite the risk of infection. This confirms the results of a study by Harries et al., in which participants stated that they would like to return to clinical rotations regardless of the risk of infection [9]. In relation to the lectures, however, the students rated face-to-face attendance to the contrary and stated that they would like to keep the digital courses (e-learning programs and digital lectures) in future. Overall, the participants confirmed that the online courses motivated them to study more. This confirms the results of other studies indicating high motivation due to e-learning [10–12]. Although most students indicated that they would prefer to further reduce face-to-face events and replace them with online courses, a minority negated this, as they preferred face-to-face teaching.

From a technical point of view, the changeover went well at our institution, as there were rarely technical problems with the online events. Although most students had no prior experience with online courses, they successfully participated in most of the online events. The participants in this study stated that the digitalization of dental education itself was not a stressful factor for them. Rather the opposite, as the students indicated that effective knowledge gain increased with online teaching compared to face-to-face teaching. However, it is clear that this aspect depends on the faculty’s readiness and expertise to employ technology to facilitate the learning process. In a study by Khalil et al., there were several technical problems, including poor internet connectivity or educators’ deficient basic computer skills, which caused stress for the students as the learning goals could not be achieved [13].

In addition, several studies have indicated that, apart from digitalization, the pandemic itself has been stressful for dental students [14–16]. For example, Agius et al. reported that the pandemic caused a fear of losing manual dexterity skills and influenced examinations [14]. However, the participants of our study stated that most students were not concerned that the marks of their final examination might be influenced by the pandemic. The responses to the concerns that single courses might not be passed due to the pandemic were rather ambivalent. Regarding the overall impact of the pandemic on the students’ education, the participants also had very divergent attitudes. While some rated the influence positively, others stated that the influence was rather negative. These differences can have many different causes. It certainly depends on the type of learner, the stage of training, and social circumstances. In a study by Klaassen et al., similar results were found, as about half of their participants were extremely concerned about the impact of COVID-19 on their education while the other half worried less [15]. The statement, that the pandemic made it easier for the students to focus on their studies as there was less distraction from leisure activities was negated in the present study.

In this study, students stated that the pandemic had a somewhat positive influence on the acquisition of theoretical skills and a somewhat negative influence on the acquisition of practical skills. In a practical profession like dentistry, the acquisition of practical skills is extremely important. Possible solutions to overcome the lack of practical training could be virtual simulation technologies or computer-based models of real-life processes. The advantage of such methods is having a controlled setting in a safe environment and the exclusion of risk to patients [13, 17]. Despite these digital solutions, a resumption of the courses needs to be considered under appropriate hygiene conditions [18, 19].

From the teachers_’_ point of view, the teaching atmosphere has changed a lot over the past year. At our department, the lecturers agree that the online courses were a good solution to continue teaching during the pandemic. Still, teaching in online courses has become impersonal and the interaction between the lecturer und the students has decreased. Nevertheless, thanks to the e-learning material, the students seem to be better prepared for the lectures and internships. The teachers in our department therefore agree that classroom teaching cannot be replaced by digital formats, but that e-learning is a perfect tool to support the courses in a blended learning concept.

The major limitation of this study is that the cross-sectional survey took place only at a single institution. Further studies including multiple institutions are needed, although the comparison might be difficult as the stage of digitalization is different at each university.

Digitalization of teaching oral and maxillofacial surgery in dental education is possible but depends on the institutions preparatory work and technological possibilities. Thanks to the comprehensive blended learning offer created beforehand, our department was well prepared for the pandemic. In future, cooperation between universities should increase so that more institutes can benefit from the digitalization that has taken place at single institutions. Students declared a high acceptance of the digital learning formats and indicated an increased motivation to learn due to the e-learning materials. The pandemic influence on the students’ education was rated ambivalent.

## Supplementary Information

**Additional file 1.**

## Data Availability

The datasets used and/or analysed during the current study are available from the corresponding author on reasonable request.
