# Supplementary material for: One year of COVID-19 pandemic: a cross sectional study on teaching oral and maxillofacial surgery
Source: Head Face Med. 2021 Dec 18;17:51. doi: 10.1186/s13005-021-00304-z (PMC8683806; doi:10.1186/s13005-021-00304-z)
Supplement: Supplementary file 1 — Additional file 1. [file 13005_2021_304_MOESM1_ESM.docx]

**Evaluation sheet- digital teaching during the past year**

1. Demographic and general information
   1. Gender

□ female □ male □ various

- 1. Age

□ ≤21 J. □ 22-25 J. □ ≥26 J.

- 1. Study year

□ 3^rd^ year □ 4^th^ year □ 5^th^ year □ 6^th^ year

- 1. How often did you participate on an online lecture/presentation during the past year?

□ Never □ ≤ 20% □ 21-40% □ 41-60% □ 61-80% □ ≥81%

1. Internships
   1. Were internships cancelled during the summer semester 2020?

□ yes □ no

- 1. In percentage, how many internships were cancelled during the summer semester 2020?

□ ≤ 25% □ 26-50% □ 51-75% □ ≥76%

- 1. Were the cancelled internships replaced or caught up on?

□ yes □ no □ partially

- 1. Were internships cancelled during the winter semester 2020/2021?

□ yes □ no

- 1. In percentage, how many internships were cancelled during the winter semester 2020/2021?

□ ≤ 25% □ 26-50% □ 51-75% □ ≥76%

- 1. Were the cancelled internships replaced or caught up on?

□ yes □ no □ partially

- 1. Despite the risk of infection, I would have liked to have continued the internships normally from the start.

□1 □2 □3 □4 □5 □6 □7 □8 □9 □10

Totally agree Totally disagree

1. Lectures
   1. Were lectures cancelled during the summer semester 2020?

□ yes □ no

- 1. In percentage, how many lectures were cancelled during the summer semester 2020?

□ ≤ 25% □ 26-50% □ 51-75% □ ≥76%

- 1. Were the cancelled lectures replaced or caught up on?

□ yes □ no □ partially

- 1. Were lectures cancelled during the winter semester 2020/2021?

□ yes □ no

- 1. In percentage, how many lectures were cancelled during the winter semester 2020/2021?

□ ≤ 25% □ 26-50% □ 51-75% □ ≥76%

- 1. Were the cancelled lectures replaced or caught up on?

□ yes □ no □ partially

- 1. Despite the risk of infection, I would have liked to have continued the lectures normally from the start.

□1 □2 □3 □4 □5 □6 □7 □8 □9 □10

Totally agree Totally disagree

1. E-Learning
   1. Did you have any experience with online courses before the pandemic?

□ yes □ no

- 1. Thanks to the extensive digital range of oral and maxillofacial surgery online (Emedia Skills Lab) developed in recent years, the MKG was well prepared for the pandemic.

□1 □2 □3 □4 □5 □6 □7 □8 □9 □10

Totally agree Totally disagree

- 1. In my opinion, the effectiveness of knowledge transfer through online events has improved significantly compared to face-to-face events.

□1 □2 □3 □4 □5 □6 □7 □8 □9 □10

Totally agree Totally disagree

- 1. In the last two semesters there were often technical problems with online teaching.

□1 □2 □3 □4 □5 □6 □7 □8 □9 □10

Totally agree Totally disagree

- 1. The change from face-to-face to online teaching worked very well in the last two semesters.

□1 □2 □3 □4 □5 □6 □7 □8 □9 □10

Totally agree Totally disagree

1. Pandemic-related solutions/effects
   1. The organization of the switch from face-to-face to online teaching was very good.

□1 □2 □3 □4 □5 □6 □7 □8 □9 □10

Totally agree Totally disagree

- 1. How has the pandemic affected your student education overall?

□1 □2 □3 □4 □5 □6 □7 □8 □9 □10

Positive Negative

- 1. How has the pandemic affected the acquisition of my theoretical knowledge?

□1 □2 □3 □4 □5 □6 □7 □8 □9 □10

Positive Negative

- 1. How has the pandemic affected the acquisition of my practical skills?

□1 □2 □3 □4 □5 □6 □7 □8 □9 □10

Positive Negative

- 1. What is the impact of the pandemic on your practical training?
  2. The new digital learning conditions are very stressful for me.

□1 □2 □3 □4 □5 □6 □7 □8 □9 □10

Totally agree Totally disagree

- 1. Why are you stressing the new digital learning conditions?
  2. I am concerned that I will not pass some courses because of the pandemic conditions.

□1 □2 □3 □4 □5 □6 □7 □8 □9 □10

Totally agree Totally disagree

- 1. I am concerned that my exam grades will change due to the pandemic conditions.

□1 □2 □3 □4 □5 □6 □7 □8 □9 □10

Totally agree Totally disagree

- 1. I would like to keep the digital courses (e-learning programs and digital lectures) in the future.

□1 □2 □3 □4 □5 □6 □7 □8 □9 □10

Totally agree Totally disagree

- 1. In the future, I prefer to further reduce face-to-face events and replace them with online courses.

□1 □2 □3 □4 □5 □6 □7 □8 □9 □10

Totally agree Totally disagree

- 1. Which digital teaching format are you most likely to prefer?

□ live-lectures □ recorded lectures □ e-learning programs

- 1. The online courses motivated me to learn more.

□1 □2 □3 □4 □5 □6 □7 □8 □9 □10

Totally agree Totally disagree

- 1. The pandemic made it easier for me to focus on my studies because there was less distraction from leisure activities.

□1 □2 □3 □4 □5 □6 □7 □8 □9 □10

Totally agree Totally disagree

- 1. Do you have any further comments on the teaching of oral- and maxillofacial surgery during the pandemic?
